# Metachronous Anal Canal and Prostate Cancers with Simultaneous Definitive Therapy: A Case Report and Review of the Literature

**DOI:** 10.1155/2011/864371

**Published:** 2011-09-15

**Authors:** Edward F. Miles, Laura L. Jacimore, John W. Nelson

**Affiliations:** ^1^Division of Radiation Oncology, Department of Radiology, Naval Medical Center Portsmouth, 620 John Paul Jones Circle, Portsmouth, VA 23708, USA; ^2^Division of Radiation Oncology, Nash General Hospital, 2460 Curtis Ellis Drive, Rocky Mount, NC 27804, USA

## Abstract

Anal canal cancer is rare, accounting for only 1.3% of all gastrointestinal tract malignancies. Prostate cancer incidence is much higher and accounts for 27.6% of all malignancies in men. Treatment guidelines for anal cancer involve radiotherapy to the primary site and draining lymphatics while treatment for prostate cancer can also include pelvic radiotherapy. The literature is silent on the optimum course of action when these two malignancies are found synchronously or metachronously. Herein, we report a case of a patient diagnosed with intermediate risk prostate cancer who, prior to definitive therapy for this first malignancy, was also diagnosed with anal canal cancer. We conclude that a simultaneous approach with radiation therapy and chemotherapy with subsequent boost to the prostate is recommended. Screening for synchronous prostate cancer in male anal canal cancer patients is probably indicated and may preclude suboptimal treatment for a second occult primary.

## 1. Introduction

Squamous cell carcinoma of the anal canal is rare and accounted for only 0.3% of the total estimated new cases of cancer in the USA in 2010. In men, this percentage was even lower at 0.25%, comprising an estimated 2,000 cases, and accounting for only 1.3% of all gastrointestinal tract malignancies. Conversely, prostate cancer incidence is more than 100 times higher and accounts for approximately 27.6% of all malignancies in men [1]. Established treatment guidelines for localized anal cancer involve radiotherapy to the primary site and draining lymphatics to include the pelvic and inguinal nodes [2] as well as the use of concurrent systemic chemotherapy [3]. The use of pelvic radiotherapy is also well established for the treatment of localized prostate cancer, with or without concurrent hormonal therapy [4]. Unfortunately, the literature is silent on the optimum course of action when these two malignancies are found synchronously, or metachronously, before definitive therapy has been initiated for either.

Herein, we report a case of a patient diagnosed with intermediate risk prostate cancer who, prior to definitive therapy for this first malignancy, was also diagnosed with early-stage anal canal cancer.

## 2. Case Presentation

**Figure 1:**
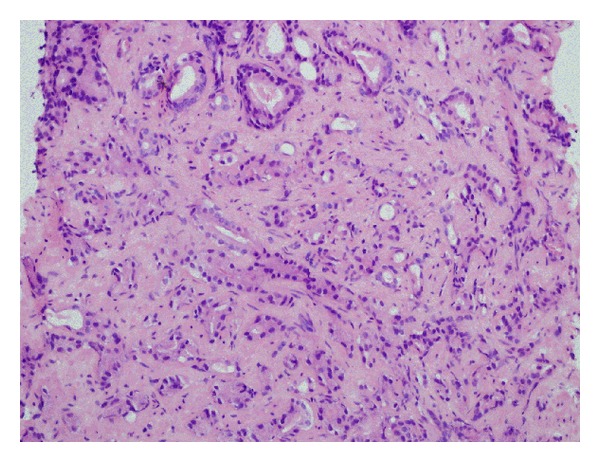
Prostate adenocarcinoma from biopsy (hematoxylin and eosin, 200x).

**Figure 2:**
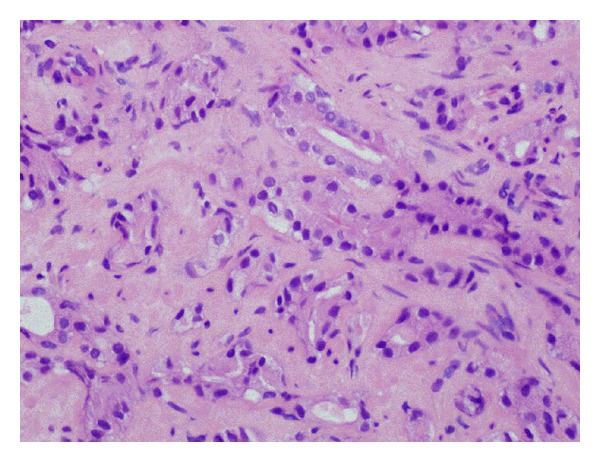
Prostate adenocarcinoma from biopsy (hematoxylin and eosin, 400x).

**Figure 3:**
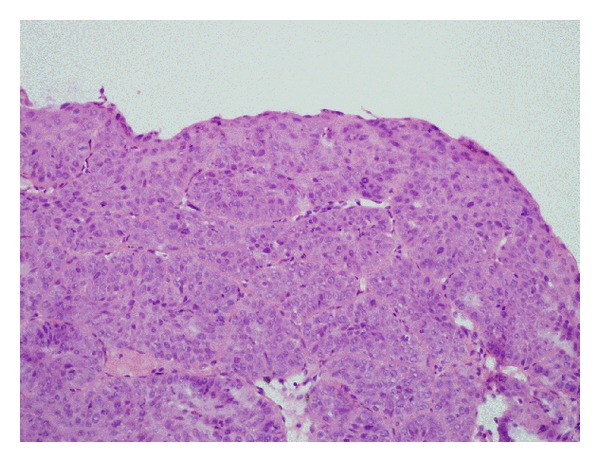
Squamous cell carcinoma in situ of the anus with underlying invasion from biopsy (hematoxylin and eosin, 200x).

The patient was a 68-year-old human-immunodeficiency virus- (HIV-) negative African-American male who presented for consideration of radiation therapy for prostate cancer: clinical stage T1c, Gleason score 4 + 3 = 7 (Figures 1 and 2), prostate specific antigen (PSA) 14.5 nanogram/deciliter. Further workup with a bone scan and a computed tomography (CT) scan of the abdomen and pelvis were negative for any evidence of metastatic disease. He was offered definitive radiation therapy, but elected instead to pursue definitive surgery. However, due to his numerous, poorly controlled comorbidities including diabetes mellitus, hypertension, renal insufficiency, gout, and coronary/peripheral artery disease, he was started on androgen deprivation therapy with the gonadotropin-releasing hormone agonist leuprolide as a temporizing maneuver while optimizing management of these conditions prior to surgery. After 18 months of hormonal monotherapy and prior to commencing his planned open radical prostatectomy, he developed scant hematochezia. Evaluation via physical exam, colonoscopy, and biopsy revealed a 1.5 centimeter invasive squamous cell carcinoma (Figure 3) at the anorectal junction. Staging studies including a positron emission tomography (PET)/CT scan showed a T1 N0 anal canal cancer, Stage I. There was no evidence of metastatic disease from either primary malignancy. He was evaluated in a multidisciplinary setting with surgical, medical, and radiation oncologists; the decision was made to treat both primaries simultaneously. 

He underwent a planning CT in the supine position. Due to the relative lack of experience at the time in treating anal canal cancer with intensity-modulated radiation therapy (IMRT), his initial plan consisted of a wide anterior-posterior (AP)/narrow posterior-anterior (PA) field arrangement to treat the primary pelvic disease and draining lymph nodes with concurrent mitomycin-C and capecitabine, a common approach for anal canal cancer treatment. Six MV photons were used with a 1-centimeter bolus material applied daily to the anterior inguinal nodal region to ensure nodal coverage to a dose of 3,060 centigray (cGy) using 180 cGy fractions. The superior border of the field was moved inferiorly from the L5/S1 interspace to the level of the true pelvis, and the dose was carried to 3,600 cGy. The lateral field borders of the AP field were then narrowed to match the divergence of the PA field at midplane, and treatment was continued to 5,040 cGy to complete the therapy for his anal canal cancer. At this point, an IMRT boost was planned to encompass the prostate and proximal seminal vesicles with margin to bring the dose for his prostate malignancy to 7,380 cGy. His treatment progressed as planned although a one-week treatment break was required due to moist desquamation in the bilateral inguinal and intergluteal areas. Otherwise, he completed the combined modality therapy with no significant gastrointestinal or genitourinary toxicity. His androgen deprivation therapy was continued during his radiation therapy and then stopped. Notwithstanding his 18 months of temporizing hormonal therapy, the hormonal therapy administered during his definitive radiation therapy could be considered to be most consistent with short-term neoadjuvant and hormonal therapy which is supported by several randomized trials for men with intermediate risk disease [5, 6]. He has been followed regularly since that time with routine physical exams (including regular anoscopy) and serum PSA checks. At his last followup, 18 months after completing simultaneous therapy for both malignancies, he has no evidence of disease recurrence of either cancer and no significant gastrointestinal or genitourinary toxicity from his therapy.

## 3. Discussion

Accepted treatment regimens for both prostate cancer and anal canal cancer are well established. However, the treatment of either malignancy with radiation therapy in isolation would likely preclude optimum future treatment of the other, either by removing further pelvic radiotherapy as an option or making definitive surgery substantially more difficult due to pelvic adhesions. 

In this case, the patient was first diagnosed with prostate cancer. The definitive therapy that he selected, radical prostatectomy, was delayed while attempting to optimize management of his multiple comorbidities. Given the severity of these conditions, consideration was given to not initiate any therapy at all for his prostate cancer, or at most, simply continuing his hormonal therapy alone which was effectively controlling his serum PSA. However, with a PSA greater than 10 and a Gleason sore of 4 + 3 = 7 in greater than 50% of the cores obtained, he was stratified as “high-intermediate” risk [7] and both the patient and the treating team considered definitive therapy important to pursue.

Had our patient initially selected definitive radiation therapy (with or without concurrent hormonal therapy) for treatment of his prostate cancer, optimal therapy for his metachronous anal canal cancer would have been compromised as additional pelvic radiotherapy would have been contraindicated; abdominoperineal resection (APR) with the associated permanent colostomy would likely have been required. Due to the markedly disparate levels of incidence of prostate and anal canal cancer, screening for anal canal cancer in a patient diagnosed with prostate cancer with anything other than a digital rectal exam (DRE) is not routinely indicated. However, especially in older men, the converse is probably not true. Current National Comprehensive Cancer Committee (NCCN) guidelines for the workup and staging of anal canal cancer do recommend a DRE and an abdominal/pelvic CT scan, but do not specifically recommend the additional screening step of a serum PSA test [2]. Had our patient's anal canal cancer been diagnosed first, his prostate cancer would not have been detected with a DRE; he was had clinical stage T1c at diagnosis. Additionally, his staging pelvic CT scan failed to show any concerning abnormalities in or around his prostate. The only indication of prostate cancer would have been his elevated serum PSA.

Terris and Wren reported their results of a single institution study in which 20 consecutive men scheduled for APR for a diagnosis of a colorectal malignancy were screened for prostate cancer with DRE and PSA prior to undergoing the APR. Six men were found to have an elevated PSA; two of these men were also noted to have an abnormal DRE. Prostate biopsy in these six men demonstrated that half of them had synchronous prostate cancer (15.8% of those screened). The authors recommended that male patients aged 50 years or older with at least a ten-year life expectancy scheduled for APR be screened for prostate cancer. This screening procedure detected a substantial percentage of men with a synchronous malignancy whose detection and/or treatment could have been hindered by the pending curative procedure for their colorectal malignancy [8]. Therefore, while not specifically recommended in the NCCN guidelines, it appears to make intuitive sense to check a serum PSA in older men with anal canal cancer to preclude initiating therapy that may impact the ability to treat a synchronous or metachronous pelvic malignancy, given the increased incidence of prostate cancer in this cohort. 

An analogous clinical scenario is the recommendation for a thorough gynecological exam including screening for cervical cancer in women diagnosed with anal canal cancer prior to the initiation of therapy, as treatment of all but the very earliest stages of this malignancy also involves pelvic radiation therapy [2]. This recommendation is based on the strong association of human papilloma virus (HPV) infection with both anal canal and cervical cancer. The data for an association between HPV infection and an increased risk of prostate cancer is mixed and controversial; examples include a recent serology investigation of men on the Prostate Cancer Prevention Trial [9] which failed to demonstrate an association, and a case-control study that screened prostate biopsy tissue DNA and RNA for the presence of HPV viral genomes that demonstrated a positive association [10].

In conclusion, anal canal cancer is a relatively rare malignancy whose optimum treatment involves pelvic radiotherapy with concurrent systemic chemotherapy. Prostate cancer is a much more common malignancy whose definitive treatment may be compromised by prior pelvic radiotherapy. As the literature is silent on the optimal treatment of synchronous or metachronous presentation of these malignancies, a simultaneous approach with pelvic radiation therapy and systemic chemotherapy with subsequent field adjustments to boost the prostate and seminal vesicles to a higher definitive dose is recommended. If available, the use of intensity-modulated radiation therapy can also be considered [11]. Screening for synchronous prostate cancer in male patients diagnosed with anal canal cancer via DRE and serum PSA is probably indicated and may preclude suboptimal treatment for a second occult primary.
